# VEGF Production Is Regulated by the AKT/ERK1/2 Signaling Pathway and Controls the Proliferation of *Toxoplasma gondii* in ARPE-19 Cells

**DOI:** 10.3389/fcimb.2020.00184

**Published:** 2020-04-28

**Authors:** Juan-Hua Quan, Hassan Ahmed Hassan Ahmed Ismail, Guang-Ho Cha, Young-Joon Jo, Fei Fei Gao, In-Wook Choi, Jia-Qi Chu, Jae-Min Yuk, Young-Ha Lee

**Affiliations:** ^1^Department of Gastroenterology, Affiliated Hospital of Guangdong Medical University, Zhanjiang, China; ^2^Communicable and Non-communicable Diseases Control Directorate, Federal Ministry of Health, Khartoum, Sudan; ^3^Department of Infection Biology and Department of Medical Science, Chungnam National University School of Medicine, Daejeon, South Korea; ^4^Department of Ophthalmology, School of Medicine, Chungnam National University, Daejeon, South Korea; ^5^Stem Cell Research and Cellular Therapy Center, Affiliated Hospital of Guangdong Medical University, Zhanjiang, China

**Keywords:** ocular toxoplasmosis, retinal pigment epithelium, vascular endothelial growth factor, PI3K/MAPK signaling pathways, *Toxoplasma gondii* proliferation

## Abstract

The retina is the primary site of *Toxoplasma gondii* infection in the eye, and choroidal neovascularization in ocular toxoplasmosis is one of the most important causes of visual impairment. Vascular endothelial growth factor (VEGF) is one of the key regulators of blood vessel development, however, little is known about the mechanisms of *T. gondii*-induced VEGF production in ocular toxoplasmosis. Here, we investigate the effect of *T. gondii* on VEGF production regulation in human retinal pigment epithelium ARPE-19 cells and attempted to unveil the underlying mechanism of this event by focusing on the interaction between parasite and the selected host intracellular signaling pathways. *T. gondii* infection increased the expression of VEGF mRNA and protein in ARPE-19 cells in parasite burden- and infection time-dependent manner. The proportional increase of VEGF upstream regulators, HIF-1α and HO-1, was also observed. *T. gondii* induced the activation of host p-AKT, p-ERK1/2, and p-p38 MAPK in ARPE-19 cells in a parasite-burden dependent manner. However, VEGF expression decreased after the pre-treatment with PI3K inhibitors (LY294002 and GDC-0941), ERK1/2 inhibitor (PD098059), and p38 MAPK inhibitor (SB203580), but not JNK inhibitor (SP600125), in a dose-dependent manner. The anti-VEGF agent bevacizumab or VEGF siRNA transfection prominently inhibited the activation of p-AKT and p-ERK1/2, but not p-p38 MAPK and JNK1/2 in *T. gondii*-infected ARPE-19 cells. Bevacizumab treatment or VEGF siRNA transfection significantly inhibited the proliferation of *T. gondii* tachyzoites in the host cell, dose-dependently, but not invasion of parasites. VEGF-receptor 2 (VEGF-R2) antagonist, SU5416, attenuated VEGF production and tachyzoite proliferation in *T. gondii*-infected ARPE-19 cells in a dose-dependent manner. Collectively, *T. gondii* prominently induces VEGF production in ARPE-19 cells, and VEGF and AKT/ERK1/2 signaling pathways mutually regulate each other in *T. gondii*-infected ARPE-19 cells, but not p38 MAPK and JNK1/2 signaling pathways. VEGF and VEGF-R2 control the parasite proliferation in *T. gondii*-infected ARPE-19 cells. From this study, we revealed the putative mechanisms for VEGF induction as well as the existence of positive feedback between VEGF and PI3K/MAPK signaling pathways in *T. gondii*-infected retinal pigment epithelium.

## Introduction

*Toxoplasma gondii* is an obligate intracellular protozoan parasite that infects one-third of the world's population (Robert-Gangneux and Dard, 2012). Infection is most commonly acquired through the ingestion of raw or undercooked meat containing the cystic bradyzoite form or through ingesting materials contaminated by cat feces that may contain oocysts (Halonen and Weiss, 2013). Almost 80–90% of primary *T*. *gondii* infections are asymptomatic in immunocompetent individuals (Halonen and Weiss, 2013); however, toxoplasmic retinochoroiditis is a progressive, recurring disease that can cause severe morbidity (Commodaro et al., 2009). In the United States, 2.0% of persons infected with *T. gondii* have ocular toxoplasmosis, and 0.45% develop symptomatic ocular toxoplasmosis (Jones and Holland, 2010); however, the pathophysiology of ocular toxoplasmosis is not well-understood, yet.

The retina is the primary site of *T. gondii* infection in the eye, and choroidal neovascularization in ocular toxoplasmosis is one of the most important causes of visual impairment (Commodaro et al., 2009). The development and homeostasis of ocular vasculature rely on multiple growth factors controlled by their respective signaling pathways, including vascular endothelial growth factor (VEGF), angiopoietin, TGF-β, NOTCH and Wnt (Dou et al., 2012; Apte et al., 2019; Wang et al., 2019). VEGF represents a growth factor with important pro-angiogenic activity, having a mitogenic and an anti-apoptotic effect on endothelial cells, increasing the vascular permeability, promoting cell migration, and so on (Ferrara, 2004; Melincovici et al., 2018; Apte et al., 2019). VEGF is expressed predominantly on vascular endothelial cells but can also be found on non-endothelial cells such as macrophages, keratinocytes, retinal pigmentary epithelial cells, bronchial epithelial cells and mast cells, and it actively contributes to the regulation the normal and pathological angiogenic processes (Ferrara, 2004; Johnzon et al., 2016; Melincovici et al., 2018). However, there is insufficient information regarding VEGF expression in *T. gondii*-infected retina.

Human retinal pigment epithelium (RPE) ARPE-19 cells are highly polarized cells that form the outer blood-retina barrier between the photoreceptors of the neurosensory retina and vascularized choroid. The RPE mediates the exchange between the choriocapillaris and the photoreceptors and secretes growth factors such as VEGF (Strauss, 2005). Thus, the RPE has been implicated in a variety of retinal diseases, and pathological angiogenesis of the retina is a key component of irreversible causes of blindness including proliferative diabetic retinopathy, age-related macular degeneration, retinopathy of prematurity and toxoplasmic retinochoroiditis (Pleyer et al., 2014; Rezzola et al., 2014).

Toxoplasmic retinochoroiditis is one of the late consequences of ocular toxoplasmosis, and choroidal neovascularization is a severe complication of ocular toxoplasmic retinochoroiditis (Commodaro et al., 2009; Pleyer et al., 2014). There is ample evidence that VEGF secretion by RPE cells leads to neovascularization in the eye (Rezzola et al., 2014; Lie et al., 2019; Mushtaq et al., 2019). Nevertheless, information regarding the mechanisms of angiogenesis in ocular toxoplasmosis is limited. Therefore, to elucidate the characteristics of *T. gondii-*induced VEGF production and identify the signaling pathways of VEGF in the retina, we evaluated the expression patterns of VEGF in relationship with parasite proliferation and the roles of PI3K/MAPK pathways as a signal transduction pathway in *T. gondii*-infected ARPE-19 cells.

## Materials and Methods

### Reagents and Antibodies

The primary antibodies against phosphorylated ERK1/2 (p-ERK1/2), total ERK1/2 (ERK1/2), p-JNK1/2, JNK1/2, p-p38 MAPK, p38 MAPK, phosphospecific AKT (p-AKT), total AKT (AKT), HIF-1α and HO-1 were purchased from Cell Signaling Technology Inc. (Danvers, MA). The PI3K inhibitors (LY294002 and GDC-0941) and VEGF-receptor 2 (VEGF-R2) antagonist (SU5416) were obtained from Sigma (St. Louis, MO). Anti-*T. gondii* p30 antibody (TP3), secondary antibodies and anti-α tubulin was purchased from Santa Cruz Biotechnology (Santa Cruz, CA). ERK1/2 inhibitor (PD98059), p38 MAPK inhibitor (SB203580) and JNK1/2 inhibitor (SP600125) were purchased from Calbiochem (San Diego, CA). Anti-VEGF agent bevacizumab (Avastin^TM^) was obtained from Roche Korea (Diagnostics Korea, Korea).

### Cell Line and Parasites

The human ARPE-19 cell line was purchased from the ATCC (Manassas, VA). The cells were routinely grown in DMEM/F12 medium (WelGENE, Korea) supplemented with 10% heat-inactivated fetal bovine serum (FBS; Gibco BRL, Grand Island, NY), 2 mM glutamine, 100 U/mL penicillin and 100 μg/mL streptomycin. The cells were cultured at 37°C in 5% CO_2_, and passaged every 3–4 days. The viability of the ARPE-19 cells was assessed by staining with trypan blue dye.

The RH tachyzoites of *T. gondii* expressing GFP or RFP were maintained by *in vitro* ARPE-19 cells at 37°C in 5% CO_2_. After spontaneous host cell rupture, parasites and cellular debris were pelleted by centrifugation and washed with cold PBS. The final pellet was resuspended and passed through a 26-gauge needle and finally filtered through 5.0-μm pore sized filter (Millipore, Billerica, MA) to obtain a tachyzoite suspension free of host cell debris.

### *T. gondii* Infection in Mice

Murine model of ocular toxoplasmosis was prepared according to the previous reports (Dukaczewska et al., 2015; Song et al., 2018). The Me49 strain of *T. gondii* was maintained and infected in female Balb/c mice (Daehan Biolink, Chungbuk, Korea) following oral administration of 20 cysts. Every week, five mice were sacrificed for the determination of VEGF expression in the eyeball. The eyeballs from uninfected mice were removed in parallel and an equal volume of saline was orally administered as a control.

### Experimental Design

ARPE-19 cells were seeded into 96-well plates (for MTT assay), 24-well plate (for ELISA), 12-well plate (for *T. gondii* infection rate or proliferation assay), or 60 mm culture dishes (for western blot) at various densities and grown to confluence at 37°C in 5% CO_2_.

VEGF expressions were evaluated in *T. gondii*-infected ARPE-19 cells after treatment with or without specific PI3K or MAPK inhibitors using qRT-PCR, ELISA or western blot. We also examined the expressions of heme oxygenase-1 (HO-1) and hypoxia-inducible factor-1α (HIF-1α) as upstream regulators of VEGF by western blot (Dukaczewska et al., 2015; Song et al., 2018). Additionally, mice were infected with *T. gondii* Me49 strains orally and the VEGF expression in the eyeballs evaluated serially. To investigate the role of the PI3K/MAPK signaling pathways in VEGF production, ARPE-19 cells were pretreated with or without an anti-VEGF agent and infected with *T. gondii*, then the expressions of p-AKT, p-ERK1/2, p-p38 MAPK and p-JNK1/2 were evaluated by western blot. To evaluate the cytotoxicity of the anti-VEGF agent and PI3K/MAPK inhibitors, ARPE-19 cells and *T. gondii* tachyzoites were incubated with various concentration bevacizumab (BCM) or inhibitors and viability evaluated by MTT assay. To investigate the effects of an anti-VEGF agent on *T. gondii* proliferation, ARPE-19 cells were preincubated with bevacizumab or VEFG knockdown cells were prepared using VEGF specific siRNA. To evaluate the roles of autocrine VEGF signaling pathway in regulation of PI3K/ERK1/2 pathway and *T. gondii* proliferation *T. gondii-*infected AREP-19 cells, ARPE-19 cells were pre-incubated with inhibitors of VEGF-R2, AKT and ERK1/2 for 1 h and then infected with *T. gondii*. Each experiment was performed at least in triplicate.

### MTT Assay for Cell Viability

Cell viability was determined using the MTT assay. ARPE-19 cells or live *T. gondii* tachyzoites were seeded into 96-well plates (1 × 10^4^/well). PI3K/MAPK inhibitors or anti-VEGF agent were added to each well, and the plates were incubated for 24 or 48 h. MTT solution (10 μL, 5 mg/mL) was then added, and the ARPE-19 cells or *T. gondii* tachyzoites were incubated at 37°C for a further 4 h. Following the removal of the MTT solution, water-insoluble formazan was dissolved by adding 100 μL of dimethyl sulfoxide (DMSO) to each well. Finally, optical density (OD) at 570 nm was measured using a microplate reader (Sunnyvale, CA). Data shown were calculated as follows: cell viability (%) = A570 (experimental group) × 100/A570 (control).

### Immunofluorescence Microscopy

ARPE-19 cells were seeded on coverslips in 12-well plates at a density of 1 × 10^4^ cells/well and incubated for 24 h. Subsequently, ARPE-19 cells were infected with *T. gondii* tachyzoites at a multiplicity of infection (MOI) of 0, 1, 5, and 10 for 24 h or at an MOI of 10 for 0.5, 1, 18, and 24 h. Then, the coverslips were washed with PBS and fixed with 4% formaldehyde. Cells were washed five times with PBS containing 0.3% Triton X-100 (PBS-T) for 10 min and incubated with the anti α-Tubulin antibody for 2 h at room temperature. Cells were washed to remove the excess primary antibody and incubated with the appropriate fluorescently-labeled anti-mouse Alexa Fluor 488 for 2 h at room temperature.

After mounting with VECTASHIELD HardSet antifade mounting medium with DAPI (Vector laboratoriexs), fluorescent images were acquired using a Leica confocal microscope (Leica TCS SP8). The microscope was equipped with an argon ion laser, providing excitation in the 351–364 nm range. The magnification of images were 400x. Data analysis was performed with Leica confocal software (LCS Lite; Leica Microsystems).

### Bevacizumab (BCM) Administration

ARPE-19 cells were seeded in 12-well plates (1 × 10^4^ /well) containing 18 mm glass coverslips. The plates were incubated at 37°C in 5% CO_2_ for 24 h to allow cells to adhere. BCM (0.04, 0.2 or 1.0 mg/mL) was added to each well. After 24 h incubation, cells were infected with *T. gondii* tachyzoites at an MOI of 5 for 2 h and then free, or uninfected *T. gondii* tachyzoites were washed off. After 24 h of incubation, the coverslips were washed with PBS and fixed with 4% formaldehyde. Cells were stained with Texas Redh Tephalloidin (Life Technologies Corporation, Carlsbad, CA) to label F-actin and mounted on slides using a mounting medium with DAPI (Vector Laboratories, Burlingame, CA). Next, cells were imaged using fluorescence microscopy. 100 cells in each preparation were randomly selected, and the percentages of infected cells determined to calculate the ARPE-19 cell infection rate. The *T. gondii* proliferation rate in each infected cell was evaluated by counting the number of tachyzoites in each parasitophorous vacuole in infected cells.

### siRNA Transfection

Cells were transfected with the siRNA specific for human VEGF, using Lipofectamine RNAiMAX (Life Technologies Corporation) according to the manufacturer's protocol. Cells were seeded in 6-well plates, grown for 24 h (70% confluence), and then transfected with 20 nM VEGF-specific siRNA or negative control siRNA (Santa Cruz, CA) for 48 h. Then, cells were infected with *T. gondii* for 24 h, and the knockdown efficiency was determined by qRT-PCR and western blot.

### Preparation of Nuclear and Cytosolic Fractions

Nuclear or cytosolic extracts of ARPE-19 cells were prepared using a nuclear extract kit (Active Motif, Carlsbad, CA) according to the manufacturerturer, CA). Next, Fibrillarin and anti-α-Tubulin were used as loading controls for the nuclear and cytoplasmic proteins, respectively.

### Western Blot

ARPE-19 cells were infected with *T. gondii* under various conditions and harvested. Following the PBS wash, proteins were extracted using the PRO-PREP Protein Extraction Solution (iNtRON Biotechnology, Korea) and incubated with a complete protease inhibitor cocktail (Roche, Switzerland) for 30 min on ice. After centrifugation at 14,000 *g* for 15 min at 4 Korea) and incubated with a complete protease inhibitor cocktail (Roche, Switzerland) separated by SDS-PAGE gel electrophoresis and transferred to a PVDF membrane. After blocking with 5% skimmed milk, membranes were incubated overnight at 4°C with the primary antibodies diluted in TBST supplemented with 5% BSA. Primary antibodies against the following factors were used: p-ERK1/2, ERK1/2, p-p38 MAPK, p38 MAPK, p-JNK1/2, JNK1/2, p-AKT, AKT, HIF-1α, HO-1, VEGF, β-Tubulin, α-Tubulin, and Fibrillarin. Following three consecutive washes in TBST, membranes were incubated for 90 min with HRP-conjugated anti-mouse or anti-rabbit IgG. The membrane was soaked with Immobilon Western Chemiluminescent HRP Substrate (Merck Millipore, Billerica, MA), and chemiluminescence was detected by a Fusion Solo System (Vilber Lourmat, France). The band intensities were quantified using ImageJ software (NIH, Bethesda, Maryland). The results were normalized to α-Tubulin protein levels and were expressed as fold changes over the mock-infection control group.

### ELISA

The VEGF content of the cell culture supernatant was measured using the Quantikine Human VEGF Immunoassay (R&D Systems, Inc., Minneapolis, MN) according to the manufacturer's protocol. The ARPE-19 cells were seeded into 24-well culture plates (1.0 × 10^4^ cells/well) and incubated for 24 h. ARPE-19 cells were infected with *T. gondii* at a MOI of 1, 5 and 10 with or without BCM (0.04, 0.2 or 1.0 mg/mL), inhibitors of PI3K (0.1, 1, or 10 μM LY294002; 2.5, 25, or 250 nM GDC-0941), MAPKs (PD98059 0.3, 3, or 30 μM; SB203580 0.3, 3, or 30 μM; SP600125 0.3, 3, or 30 μM) and VEGF-R2 antagonist (0, 100, 500, 2500 nM RU5416). Then, the supernatant was collected at 24 and 48 h and stored at −70°C until use. VEGF levels were calculated based on standard curves generated using 15.6–1,000 pg/mL recombinant VEGF165. All assays were performed in triplicate.

### Quantitative Real-Time Polymerase Chain Reaction (qRT-PCR)

ARPE-19 cells were cultured in 60 mm culture plates and infected with *T. gondii* with or without specific PI3K, ERK1/2, p38 MAPK, or JNK1/2 inhibitors. Total RNA was extracted using TRIzol Reagent (Invitrogen, Carlsbad, CA) and RNA was transcribed into cDNA using M-MLV reverse transcriptase (Invitrogen Life Technologies) as described by the manufacturer. qRT-PCR was performed using Power SYBR® Green PCR Master Mix (Applied Biosystems, Foster City, CA). The primers used in this study are summarized in Table 1. All reactions were performed with an ABI 7500 Fast Real-Time PCR system (Applied Biosystems, Carlsbad, CA) under the following conditions: 95°C for 10 min, followed by 40 cycles of 95°C for 15 s and 60°C for 60 s. Relative VEGF gene expression levels were quantified based on the cycle threshold (Ct) values and normalized to the reference gene glyceraldehyde-3-phosphate dehydrogenase (GAPDH) or hypoxanthine phosphoribosyltransferase 1 (HPRT1). Each sample was analyzed in triplicate, and the gene expression levels were calculated using the 2^Δ*ΔCt*^ method.

**Table 1:** Primer sequences used for qRT-PCR in the current study.

**Gene name**	**Primer sequence (5^**′**^-3^**′**^)**	**Product size (bp)**	**GenBank accession No**.
mVEGF	F-CTGCTGTAACGATGAAGCCCTG	119	NM_001025250.3
	R-GCTGTAGGAAGCTCATCTCTCC		
mGAPDH	F-AGAACATCATCCCTGCATCC	110	NM_001289726.1
	R-CACATTGGGGGTAGGAACAC		
hVEGF	F-TTGCCTTGCTGCTCTACCTCCA	126	NM_001171623.1
	R-GATGGCAGTAGCTGCGCTGATA		
hHPRT1	F-GACCAGTCAACAGGGGACAT	111	NM_000194.3
	R-CTGCATTGTTTTGCCAGTGT		

### Statistical Analysis

All assays were performed using at least three separate experiments and in triplicate. Data were expressed as the means ± standard deviation (SD). All data were analyzed using unpaired, two-tailed Student's *t*-test with Bonferroni adjustment or ANOVA for multiple comparisons. A *P*-value less than 0.05 was considered to indicate statistical significance.

## Results

### *T. gondi* Increased VEGF Production in ARPE-19 Cells in a Parasite Burden—and Infection Time-Dependent Manner

**Figure 1:**
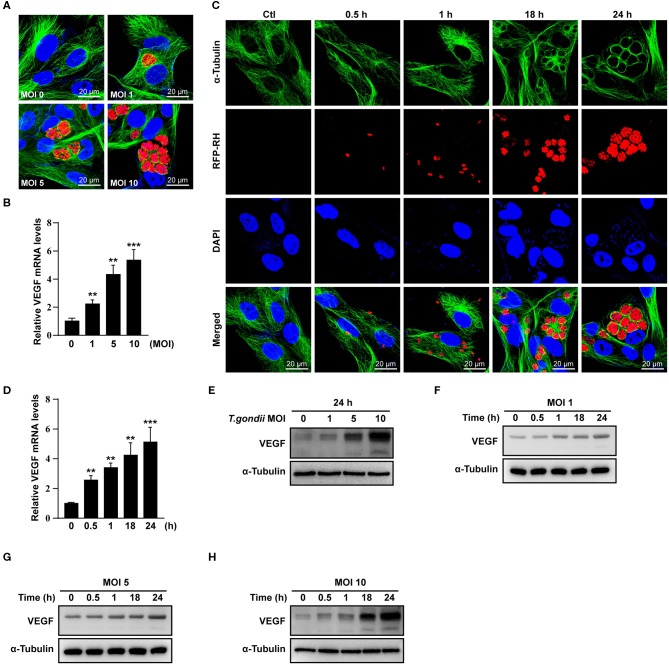
VEGF production in *T. gondii-*infected ARPE-19 cells. **(A,C)** ARPE-19 cells were infected with *T. gondii* tachyzoites for 24 h, fixed and probed against α-Tubulin. The cells were counterstained with DAPI and visualized by confocal microscope. **(B,E)** ARPE-19 cells were infected with *T. gondii* at multiplicity of infection (MOI) of 1, 5, and 10 for 24 h and VEGF mRNA levels were evaluated using qRT-PCR **(B)** and western blot **(E)**. **(D)** ARPE-19 cells were infected with *T. gondii* at MOI 10 for 0.5, 1, 18, and 24 h and the VEGF mRNA levels evaluated using qRT-PCR. Each value represents the mean ± standard deviation (SD). **P < 0.01, ***P < 0.001 compared with control ARPE-19 cells (*n* = 3). **(F–H)** ARPE-19 cells were infected with *T. gondii* at various MOIs of 1 **(F)**, 5 **(G)**, or 10 **(H)** for 0.5, 1, 18 and 24 h and the VEGF protein levels evaluated using western blot.

ARPE-19 cells were infected with *T. gondii* RH strain at MOI of 1, 5, and 10 (Figure 1A) for 24 h, and at MOI of 10 for 0.5, 1, 18, and 24 h (Figure 1C). The integrity of the microtubule network using immunofluorescence microscopy was then assessed. Visualization upon immunofluorescence staining of ARPE-19 cells by confocal microscopy displayed a well-developed array of hair-like microtubule network of slim fibrous microtubules (green) wrapped around the cell nucleus (blue) in the control cells. On the contrary, the microtubule network was reorganized around the *T. gondii* parasitophorous vacuole membrane (Figure 1C) in the infected cells.

When ARPE-19 cells were infected with *T. gondii* at an MOI of 1, 5, and 10 for 24 h, VEGF mRNA levels were significantly increased in a parasite dose-dependent manner (Figure 1B). The VEGF mRNA levels were also increased from 30 min after infection with *T. gondii* at MOI of 10 and the maximum expression of VEGF was revealed at 24 h after infection in case of 24 h investigation (Figure 1D). As shown in Figure 1E, VEGF protein levels were increased in a *T. gondii* parasite burden-dependent manner. We further evaluated the VEGF production according to the MOI and incubation time of *T. gondii* in ARPE-19 cells. VEGF expressions were tended to increase in proportion to the duration of the infection and the concentration of the parasites, thus VEGF expression was maximum at MOI 10 for 24 h postinfection when observed for 24 h (Figures 1F–H).

To investigate whether VEGF expression can be induced in non-viable parasites, we prepared heat killed (incubation at 56°C for 50 min) and formaldehyde fixed (incubation at 4% formaldehyde in PBS for 10 min) *T. gondii* tachyzoites. Unfortunately, heat killed *T. gondii* did not stimulated VEGF production in ARPE-19 cells (Supplementary Figure 1), and formaldehyde fixed tachyzoites also did not affected VEGF production (Supplementary Figure 2). These data presented that live *T. gondii* induced VEGF production in ARPE-19 cells, parasite dose- and incubation time-dependently, but not in heat killed or formaldehyde fixed tachyzoites.

### VEGF Production Was Increased in the Eyes of *T. gondii*-Infected Mice

**Figure 2:**
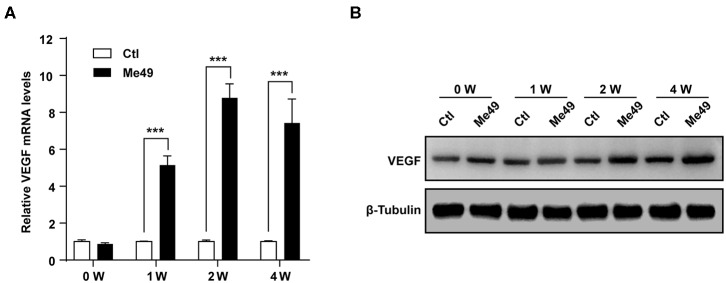
VEGF production in the eyes of *T. gondii-*infected mice. Five mice were sacrificed for the determination of VEGF expression in the eyeball by qRT-PCR **(A)** and western blot **(B)**. ***P < 0.001 compared with uninfected normal control mice (*n* = 3).

Next, we examined the VEGF production in the eye of *T. gondii*-infected mice*. T. gondii* infection significantly increased VEGF mRNA levels in the mouse eye from 1 week postinfection compared to normal control mice, and the difference was more prominent at 2 and 4 weeks after the infection (Figure 2A). In addition, the VEGF protein levels were also increased from 1 week after the infection in the eyes of *T. gondii*-infected mice compared with that of the uninfected control group (Figure 2B).

### *T. gondii* Increased the HIF-1α and HO-1 Protein Levels in ARPE-19 Cells in a Parasite Burden- and Infection Time-Dependent Manner

**Figure 3:**
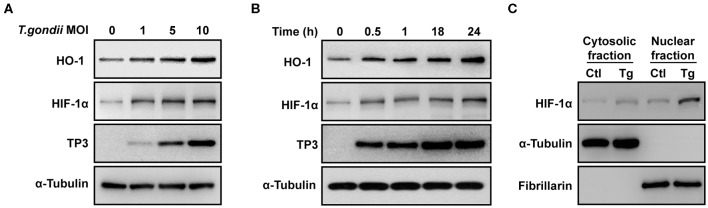
HIF-1α and HO-1 expressions in *T. gondii-*infected ARPE-19 cells. **(A,B)** ARPE-19 cells were infected with *T. gondii* for 24 h **(A)** or MOI 10 **(B)**, and protein levels were evaluated using western blot. **(C)** Nuclear and cytosolic fractions were prepared and the expression level of HIF-1α was evaluated using western blot. Anti-α-Tubulin and anti-fibrillarin were used as the cytosol and nuclear loading control, respectively. Similar results were obtained in three independent experiments (*n* = 3).

VEGF is regulated by two upstream factors, HO-1 and HIF-1α (Forooghian et al., 2007; Zhang et al., 2013). *T. gondii* infection was accompanied by the increase in the HO-1 and HIF-1α protein expression in ARPE-19 cells, which occurred in a parasite burden-dependent manner (Figure 3A). Similarly, HO-1 and HIF-1α protein levels were increased in a time-dependent manner from 30 min to 24 h (Figure 3B). Interestingly, *T. gondii* infection significantly increased the nuclear translocation of HIF-1α (Figure 3C). Taken together, these data present that *T. gondii* infection stimulated HO-1 and transcription factor HIF-1α protein expressions in ARPE-19 cells.

### PI3K Inhibitors Decreased VEGF Production in *T. gondii*-Infected ARPE-19 Cells in a Dose-Dependent Manner

**Figure 4:**
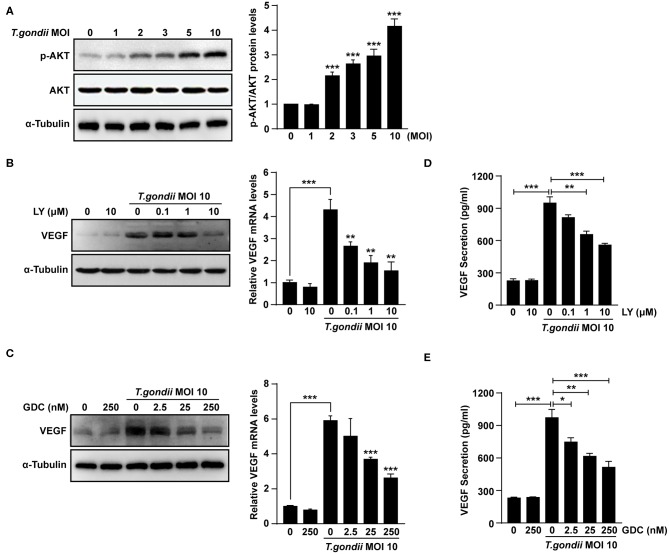
Roles of PI3K/AKT signaling pathways on *T. gondii*-induced VEGF expression in ARPE-19 cells. **(A)** ARPE-19 cells were infected with *T. gondii* at MOI 1, 2, 3, 5, and 10 for 24 h and the phosphorylation of AKT at Ser 473 was analyzed using western blot. **(B–E)** ARPE-19 cells were preincubated with the PI3K inhibitors LY294002 **(B,D)**, GDC-0941 **(C,E)** for 1 h followed by infection with *T. gondii* at MOI 10 for a further 23 h and the VEGF levels were evaluated by western blot and qRT-PCR **(B,C)** and ELISA **(E)**, respectively. *P < 0.05, **P < 0.01, ***P < 0.001 compared with control or *T. gondii*-infected cells (*n* = 3).

It was reported that VEGF production is regulated by the PI3K/AKT and MAPK signaling pathways (Klettner et al., 2013; Di et al., 2015; Meta et al., 2017). As shown in Figure 4A, levels of AKT phosphorylation at Ser473 were increased significantly following *T. gondii* infection at MOI of 1, 2, 3, 5, and 10. However, unphosphorylated total AKT levels did not change significantly after *T. gondii* infection. Next, ARPE-19 cells were treated with specific inhibitors of the PI3K pathway [LY294002 (LY) and GDC-0941 (GDC)] and VEGF production was evaluated. The cytotoxic effects of PI3K inhibitors on the viability of ARPE-19 cells were evaluated by the MTT assay. The viability of ARPE-19 cells was not affected significantly by PI3K inhibitors at the indicated concentrations (Supplementary Figure 3). After the pre-treatment with LY or GDC, VEGF mRNA and protein levels were inhibited in ARPE-19 cells infected with *T. gondii* at MOI of 10 in a dose-dependent manner in comparison to inhibitor untreated *T. gondii*-infected cells (Figures 4B,C). After the pre-treatment with 10 μM LY or 250 nM GDC at *T. gondii*-infected cells, the VEGF mRNA and protein levels almost returned to the basal levels. As shown in Figures 4D,E, VEGF secretion was also confirmed by ELISA; PI3K inhibitors resulted in a dose-dependent decrease in VEGF secretion in *T. gondii*-infected ARPE-19 cells. Therefore, the PI3K/AKT signaling pathway positively modulates the VEGF production induced by *T. gondii* infection in ARPE-19 cells.

### *T. gondii*-Induced VEGF Production in ARPE-19 Cells Was Strongly Associated With the Activation of ERK1/2 and p38 MAPK, but Not JNK1/2 Signaling

**Figure 5:**
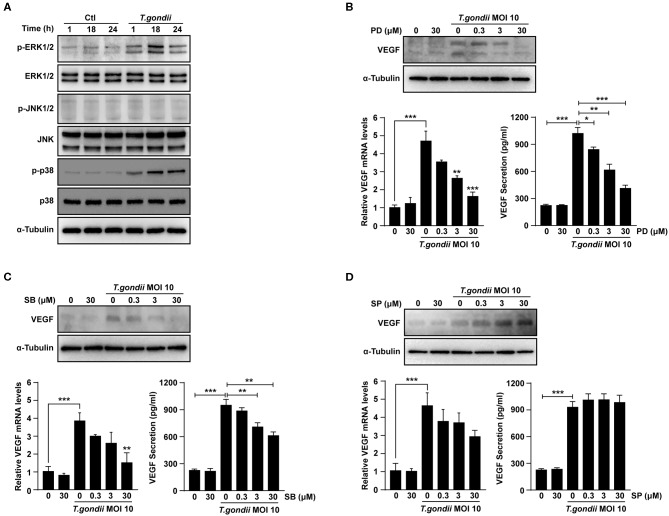
Roles of MAPK signaling pathways in *T. gondii*-induced VEGF expression in ARPE-19 cells. **(A)** ARPE-19 cells were infected with *T. gondii* at MOI 10 for 24 h and the activation of MAPK subsets were evaluated at the indicated time points. **(B–D)** ARPE-19 cells were preincubated with the PD098059 **(B)**, SB203580 **(C)** and SP600125 **(D)** for 1 h and infected with *T. gondii* MOI 10 for a further 23 h. The VEGF mRNA, protein levels and secretion levels were then evaluated by qRT-PCR, western blot and ELISA respectively. Anti-α-Tubulin was used as the internal control. Similar results were obtained in three independent experiments. *P < 0.05, **P < 0.01, ***P < 0.001 compared with control or *T. gondii*-infected cells (*n* = 3).

We evaluated the role of the MAPK pathway in *T. gondii*-induced VEGF production (Klettner et al., 2013; Di et al., 2015; Meta et al., 2017). As shown in Figure 5A, *T. gondii* infection induced an increase of p-ERK1/2 and p-p38 MAPK levels; however, p-JNK1/2 levels were not changed significantly compared to that of the control. The total protein levels of ERK1/2, p38 MAPK and JNK1/2 were not changed significantly after *T. gondii* infection.

VEGF mRNA and protein levels were examined in ARPE-19 cells which pre-treated with selective MAPK inhibitors. At the indicated concentrations of MAPK inhibitors, the viability of ARPE-19 cells was not changed significantly as compared to the control (Supplementary Figure 3). After pre-treatment with PD98059 and SB203580, the VEGF mRNA and protein levels in *T. gondii*-infected ARPE-19 cells were significantly suppressed in a dose-dependent manner than those of inhibitor untreated *T. gondii*-infected cells (Figures 5B,C). However, the VEGF levels of the inhibitor untreated *T. gondii*-infected cells were similar to those in the *T. gondii*-infected group after pre-treatment with SP600125 (Figure 5D). These results indicated that *T. gondii*-induced VEGF production in ARPE-19 cells was strongly associated with the activation of the ERK1/2 and p38 MAPK signaling pathways, but not the JNK1/2 pathway.

### VEGF and AKT/ERK1/2 Signaling Pathways Mutually Control Each Other in *T. gondii*-infected ARPE-19 Cells, but Not p38 MAPK and JNK1/2

**Figure 6:**
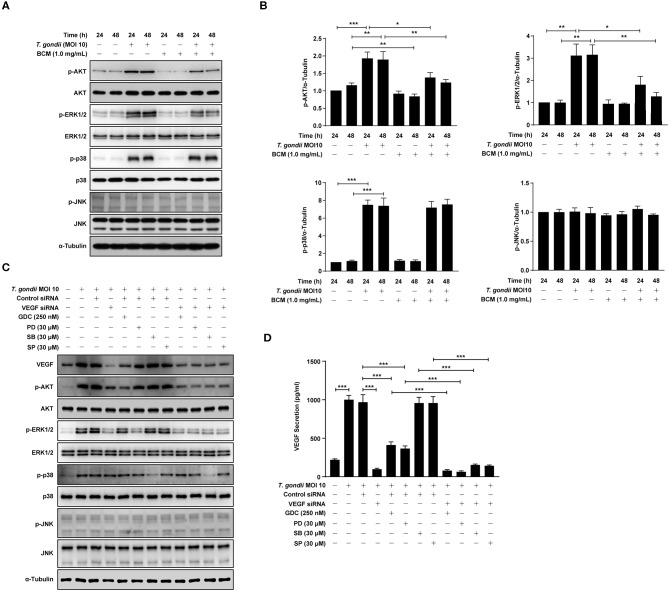
Roles of VEGF in activation of PI3K/MAPK signaling pathways in *T. gondii*-infected ARPE-19 cells. **(A,B)** ARPE-19 cells were preincubated with anti-VEGF agent bevacizumab (BCM) for 24 or 48 h and then infected with *T. gondii* at MOI 10 for 24 h. Cells were lysed and the PI3K/MAPK phosphorylation levels were assessed using western blot analysis **(A)** and quantified **(B)**. Bar plot depicting the p-pAKT/α-Tubulin, p-ERK1/2/α-Tubulin, p-p38/α-Tubulin, and p-JNK/α-Tubulin ratios as determined by densitometric analysis of western blot and expressed as fold change compared with each indicated control group. For all panels, data are presented as the mean ± SD. *P < 0.05, **P < 0.01, ***P < 0.001 compared with each indicated control group. **(C,D)** ARPE-19 cells were transfected with VEGF-specific siRNA for 48 h. ARPE-19 cells were preincubated with the GDC-0941, PD098059, SB203580, and SP600125 for 1 h and infected with *T. gondii* MOI 10 for a further 23 h. **(C)** Cells were lysed and the PI3K/MAPK phosphorylation levels were assessed using western blot analysis. Anti-α-Tubulin was used as the internal control. **(D)** The VEGF secretion levels were evaluated by ELISA. Similar results were obtained in three independent experiments. *P < 0.05, **P < 0.01, ***P < 0.001 compared with control or *T. gondii*-infected cells (*n* = 3).

In order to further evaluate the relationship between PI3K/MAPK pathway and VEGF production in *T. gondii*-infected ARPE-19 cells, cells were pre-treated with the anti-VEGF agent, BCM, and the phosphorylation of AKT, ERK1/2, p38 MAPK, and JNK1/2 was evaluated. The viability of ARPE-19 cells or *T. gondii* at the indicated concentrations of BCM were similar to those of the control group (Supplementary Figure 4). As shown in Figures 6A,B, by the pre-treatment with BCM in uninfected cells, p-AKT and p-ERK1/2 expressions were almost abolished; however, p-p38 MAPK expression was similar to that in the uninfected control group. Moreover, the level of p-AKT, p-ERK1/2 and p-JNK1/2, but not p-p38 MAPK, were decreased in *T. gondii*-infected cells by BCM pre-treatment as compared to the BMC untreated *T. gondii*-infected group. The level of whole AKT, ERK1/2, p38 MAPK, and JNK1/2 protein levels were not changed significantly by the pre-treatment with BCM in *T. gondii*-infected cells.

In order to evaluate the potential autocrine role of VEGF in amplification of PI3K/MAPK pathway and, consequently, VEGF production, VEGF siRNA transfected ARPE-19 cells were preincubated with the PI3K or MAPK inhibitors, and then infected with *T. gondii* to monitor the VEGF, AKT and MAPK subset expressions. As shown in Figure 6C, VEGF siRNA significantly inhibited the *T. gondii-* induced VEGF expression, and phosphorylation of Akt and ERK1/2 in the host cell, but phosphorylation of p38 MAPK and JNK was not affected. Alternatively, the effects of selected kinase specific inhibitors were tested on VEGF expression and the results showed that PI3K inhibitors and ERK inhibitor have significantly inhibited the *T. gondii-* induced VEGF expression in ARPE-19 cell, but p38 MAPK and JNK inhibitors did not show significant inhibition. Addition of *T. gondii*-infected VEGF siRNA to the specific kinase inhibitors treated groups showed similar or a little low VEGF expression and MAPK subset phosphorylation as compared with those of *T. gondii*-infected VEGF siRNA group. These observation implies that VEGF stimulation of host cell has a role as a foundation for *T*. *gondii-*induced AKT and ERK1/2 phosphorylation. No significant change was observed in the whole protein level of AKT, ERK1/2, p38 MAPK, and JNK1/2 by the pre-treatment with GDC, PD098059, SB203580, and SP600125.

Similar results were obtained from the ELISA which measured the secreted VEGF from the infected host (Figure 6D). Pretreatment with GDC or PD098059 showed significant inhibition of VEGF secretion from the *T. gondii*-infected group (with control siRNA transfection), but SB203580 or SP600125 pretreatment had no effect on it. When VEGF silencing was added to the condition, the VEGF level in media was reduced to the basal level. Because of that, addition of kinase inhibitors didn't have any significant effect, and this observation is in consistent with the pattern of VEGF expression and, AKT and ERK1/2 phosphorylation data.

Taken together, VEGF controls the phosphorylation of AKT and ERK1/2 in *T. gondii*-infected ARPE-19 cells. AKT and ERK1/2 signaling pathways regulate the VEGF secretion in *T. gondii*-infected ARPE-19 cells, but not p38 MAPK and JNK1/2 signaling pathways. These results imply that presence of positive feedback mechanism for VEGF expression and secretion based on ATK and ERK1/2 which can be manipulated by *T. gondii*.

### Bevacizumab Inhibited the Tachyzoite Proliferation in *T. gondii*-infected ARPE-19 Cells

**Figure 7:**
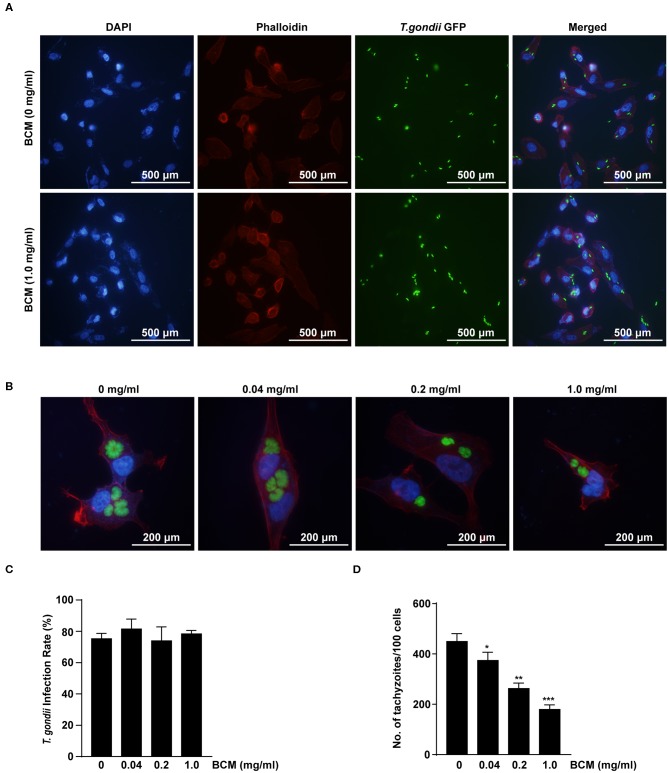
Effect of anti-VEGF agent bevacizumab on *T. gondii* invasion and proliferation in ARPE-19 cells. **(A,B)** ARPE-19 cells were treated with various doses of the bevacizumab (BCM) for 24 h and infected with *T. gondii* for 2 h **(A)** or 24 h **(B)**. The cells were stained with Texas Red-X phalloidin (red) and DAPI (blue) to identify F-actin and nuclei, respectively. **(C)** The number of *T. gondii*-infected cells and the total number of cells were counted under a fluorescence microscope. **(D)** ARPE-19 cells were pretreated with various doses of BCM for 24 h, and infected with *T. gondii* MOI 5 for 2 h, and then uninfected and free tachyzoites were washed off. Cells were incubated for a further 24 h and then fixed and intracellular parasite numbers were evaluated. *P < 0.05, **P < 0.01, ***P < 0.001 compared with *T. gondii*-infected control ARPE-19 cells (*n* = 3).

The effects of VEGF on the infection rate or proliferation of *T. gondii* tachyzoites in ARPE-19 cells were investigated following pre-treatment with various doses of the BCM for 24 h, and then infection with *T. gondii* for 2 h (Figure 7A) or 24 h (Figure 7B). The number of *T. gondii*-infected cells and the total number of cells were counted under a fluorescence microscope. There was no significant difference in the *T. gondii* infection rate in cells treated with different concentrations of BCM (Figure 7C). However, BCM significantly suppressed the proliferation of *T. gondii* in ARPE-19 cells concentration dependently and has potential growth-inhibitory activity (Figure 7D). These data indicated that BCM inhibited the tachyzoite proliferation in *T. gondii*-infected ARPE-19 cells, but not invasion of *T. gondii*.

### VEGF Expression Is Important for Control of *T. gondii* Proliferation

**Figure 8:**
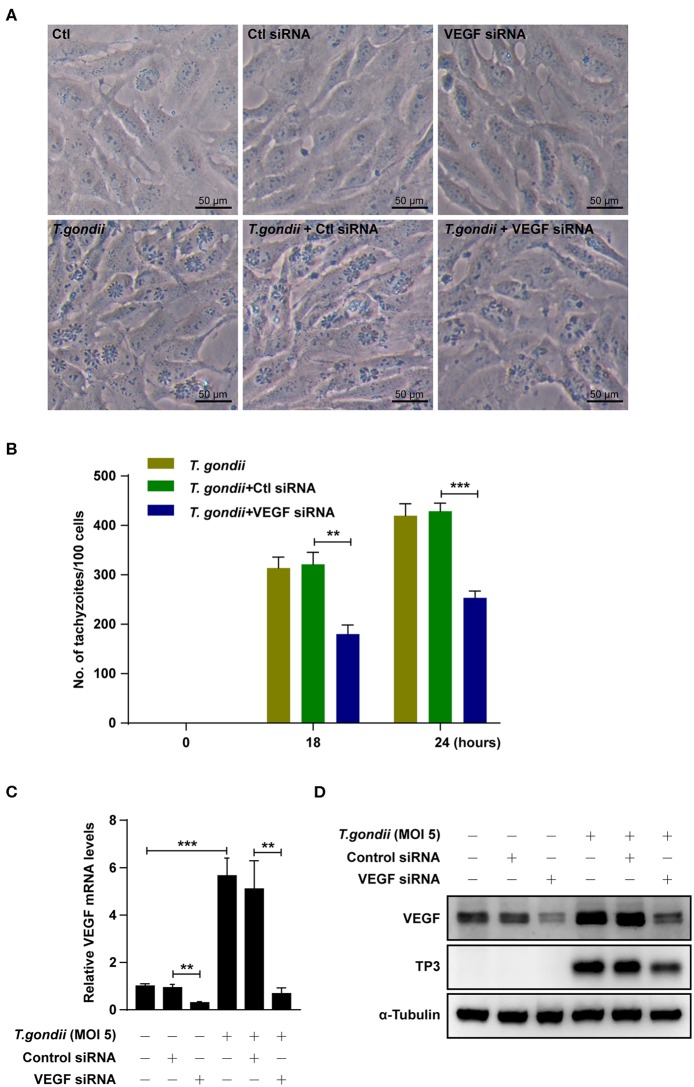
Effects of VEGF in *T. gondii* proliferation and TP3 expression. **(A)** ARPE-19 cells were transfected with VEGF-specific siRNA for 48 h and subsequently infected with *T. gondii* for 24 h. **(B)** Cells were fixed and stained with Texas Red-X phalloidin and DAPI. Intracellular GRP-RH parasites were revealed by fluorescence microscope and counted GFP parasites in 100 cells. All data shown are representative of three independent experiments. **(C)** VEGF knockdown efficiency was determined by qRT-PCR. **(D)** VEGF and *T. gondii* TP3 protein levels were detected by western blot. α-Tubulin was used as loading control. Data are representative of three independent experiments. **P < 0.01, ***P < 0.001 compared with control, control siRNA and/or *T. gondii*-infected cells (*n* = 3).

We further checked the role of VEGF in the intracellular replication of *T. gondii* using VEGF siRNA-transfected ARPE-19 cells. The number of *T. gondii*-infected VEGF siRNA-transfected cells were significantly suppressed than those of *T. gondii*-infected control siRNA-transfected cells at 18 h and 24 h postinfection (Figures 8A,B). VEGF siRNA significantly reduced the VEGF mRNA levels in uninfected cells compared with control siRNA. In addition, VEGF siRNA significantly reduced *T. gondii*-induced VEGF mRNA expression in comparison to *T. gondii*-infected control siRNA-transfected cells (Figure 8C). Similarly, VEGF siRNA significantly reduced VEGF protein levels compared with control siRNA. Furthermore, VEGF siRNA significantly reduced *T. gondii*-induced VEGF production and *T. gondii* TP3 protein levels. These results demonstrated that VEGF plays an important role in *T. gondii* proliferation in ARPE-19 cells (Figure 8D).

### VEGF-R2 Antagonist SU5416 Attenuated VEGF Production and Tachyzoite Proliferation in *T. gondii*-Infected ARPE-19 Cells in a Dose-Dependent Manner

**Figure 9:**
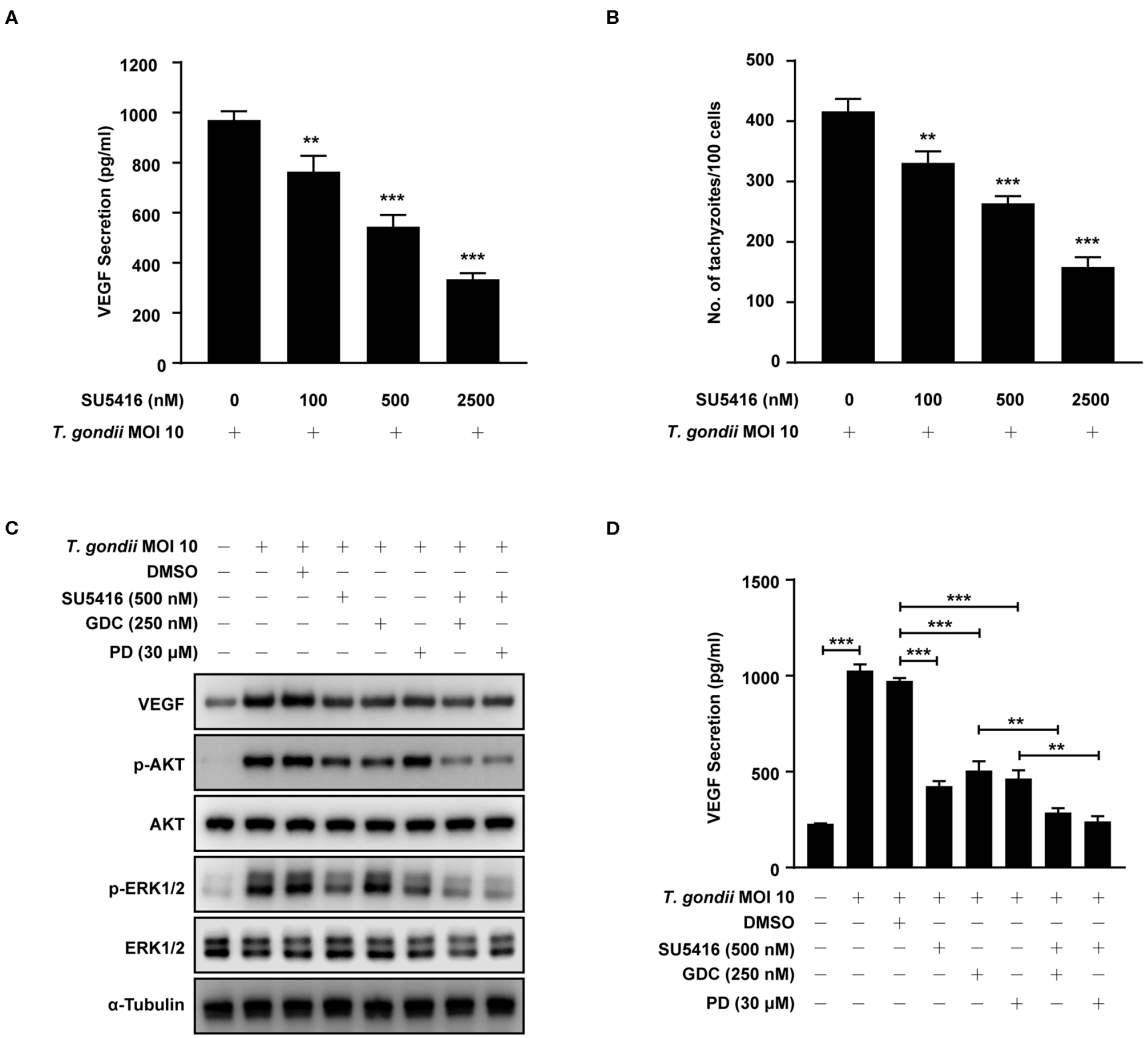
Effect of VEGF-R2 antagonist SU5416 on the regulation of VEGF production and parasite proliferation in *T. gondii*-infected ARPE-19 cells. ARPE-19 cells were pre-incubated with the indicated concentration of SU5416 for 1 h and infected with *T. gondii* MOI 10 for a further 23 h. **(A)** The VEGF secretion levels were evaluated by ELISA. **(B)** Cells were stained with Texas Red-X phalloidin and DAPI. Intracellular GFP-RH tachyzoites were revealed by fluorescence microscope and counted GFP parasites in 100 cells. ARPE-19 cells were preincubated with the DMSO, SU5416, GDC-0941, and PD098059 for 1 h and infected with *T. gondii* MOI 10 for a further 23 h. **(C)** The expression of listed proteins were determined by using western blot analysis. Anti-α-Tubulin was used as the internal control. **(D)** The VEGF secretion levels were evaluated by ELISA. Similar results were obtained in three independent experiments. **P < 0.01, ***P < 0.001 compared with indicated control cells (*n* = 3).

To evaluate the autocrine roles of VEGF signaling pathway in *T. gondii-*infected ARPE-19 cells, we prepared SU5416, a known tyrosine kinase receptor inhibitor and especially for VEGF-receptor 2 (VEGF-R2), and tested its effect on VEGF production and parasite proliferation. As shown in Figures 9A,B, SU5416 pretreatment significantly attenuated VEGF production and parasite proliferation in *T. gondii* infected ARPE-19 cells in a dose dependent manner. Together, these results demonstrate that VEGF-R2 has its autocrine regulatory role for both VEGF production and *T. gondii* proliferation in *T. gondii*-infected ARPE-19 cells.

Next, we evaluated the involvement of PI3K/ERK1/2 pathway in this autocrine VEGF signaling pathway in *T. gondii*-infected ARPE-19 cells. ARPE-19 cells were pre-incubated with the combination of DMSO (control), SU5416 (VEGF-R2 inhibitor), GDC-0941 or PD098059 (PI3K inhibitors) and then subjected to the *T. gondii* infection. Even in the presence of *T. gondii*, SU5416 pretreated group significantly reduced host VEGF protein expression and AKT and ERK1/2 phosphorylation in the host compared to the DMSO solvent control group. In addition, combined treatment of SU5416 with PI3K or ERK1/2 inhibitors showed even more augmented inhibitory effect of SU5416 against host VEGF expression and AKT and ERK1/2 phosphorylation (Figure 9C). VEGF ELISA with cell-cultured media also showed similar results that pretreatment of SU5416, GDC or PD098059 significantly inhibited VEGF level in culture media (secreted VEGF) from *T. gondii*-infected host cells (Figure 9D). These results, again, support our hypothesis and observations that VEGF-R2 signaling is, indeed, involved in regulation of AKT and ERK1/2 signaling pathways in *T. gondii*-infected ARPE-19 cells.

## Discussion

Our main finding is that *T. gondii* induced the expression of VEGF and its upstream regulators HO-1 and HIF-1α in ARPE-19 in a parasite burden- and incubation time-dependent manner. *T. gondii* activated the parasite-burden dependent phosphorylation of AKT, ERK1/2, and p38 MAPK in ARPE-19 cells, but not that of JNK1/2. Pre-treatment of PI3K, ERK1/2, p38 MAPK, and VEGR-R2 inhibitors suppressed the VEGF expression in *T. gondii*-infected cells in a dose dependent manner. Conversely, by the pretreatment with anti-VEGF agent (BCM) or VEGF siRNA, the levels of AKT and ERK1/2 phosphorylation were significantly reduced in *T. gondii*-infected ARPE-19 cells. but the identical VEGF inhibition measures had no significant effect on phosphorylation levels of the p38 MAPK and JNK1/2. Antagonists of VEGF and VEGF-R2 inhibited the proliferation of *T. gondii* tachyzoites in the host cell, dose-dependently in ARPE-19 cells, but does not control the invasion of parasites into the host cells. From this study, we revealed the putative mechanisms for VEGF induction as well as the existence of positive feedback between VEGF and PI3K/MAPK signaling pathways in RPE, which all can be stimulated by *T. gondii* infection. These findings may contribute to the understanding of the pathophysiology of ocular toxoplasmosis.

Angiogenesis is essential in the embryonic development, organ hemostasis and disease progression. Several molecules, such as VEGF, TGF-β, and angiopoietin, are known to be the key regulators of blood vessel development (Apte et al., 2019; Wang et al., 2019). VEGF is constitutively expressed in RPE to maintain physiological functions of the choroid and the RPE, however overexpression of VEGF can cause vascular disease in the retina of the eye and other parts of the body such as retinal edema, choroidal neovascularization and retinopathy of prematurity (Smith et al., 2004; Wiertz et al., 2010; de-la-Torre et al., 2014; Lie et al., 2019; Mushtaq et al., 2019). The secretion of VEGF from RPE cells is induced by physical and chemical factors such as hypoxia, advanced glycation end products (AGEs), hyperthermia, epithelial membrane protein 2 (Emp2) and cytokines (Treins et al., 2001; Faby et al., 2014; Apte et al., 2019; Wang et al., 2019; Sun et al., 2020). VEGF signaling is also regulated by endocytosis and intracellular trafficking of VEGF receptors (Eichmann and Simons, 2012). However, the mechanisms of neovascularization in ocular toxoplasmosis is not known well. In the present study, VEGF was constitutively expressed in uninfected human RPE cell line (ARPE-19 cell), and its mRNA expression was increased after *T. gondii* infection in a parasite burden- and infection time-dependent manner. Further, VEGF protein levels in the eye were notably increased in mice infected with *T. gondii*. These results are related to previous reports that VEGF plays a critical role in the survival and maintenance of RPE integrity (Ford et al., 2011) and high level of VEGF is related to the higher number of active ocular toxoplasmosis (Wiertz et al., 2010; de-la-Torre et al., 2014).

The expression of VEGF is regulated via a various factors, including external factors, signal transduction molecules, and transcription factors (Ferrara, 2004; Klettner et al., 2013). HIF-1 has been reported to be a key regulator of angiogenesis. Hypoxia increases VEGF expression by formation of HIF-1, a heterodimer consisting of HIF-1α and HIF-1β subunits, and then binding of this HIF1 dimer to the hypoxia-responsive element (HRE) in VEGF promoter (Forooghian et al., 2007; Hashimoto and Shibasaki, 2015). HO-1 is widely distributed and contributes to the formation of blood vessels both directly, through enhancing the angiogenic activities of endothelial cells, and indirectly, through regulating VEGF expression *in vivo* and *in vitro* (Bussolati and Mason, 2006; Zhang et al., 2013). In this study, *T. gondii* infection increased HIF-1α and HO-1 production in a parasite burden- and incubation time-dependent manner and the expression patterns of both genes were in positive correlation with the VEGF expression pattern after *T. gondii* infection in ARPE-19 cells. These results are consistent with the study, which reported that advanced glycation end products (AGEs), generated under hyperglycemic conditions, stimulate VEGF expression through the accumulation and the subsequent activation of HIF-1α (Treins et al., 2001).

The exact pathway responsible for the induced-VEGF secretion is not clear yet, though it is presumed this event is highly depend on the external stimulus. Previous studies have reported that PI3K/AKT and MAPK pathways are important for the regulation of VEGF expression in the eye (Klettner et al., 2013; Di et al., 2015; Meta et al., 2017). Therefore, we also examined the roles of PI3K/AKT and MAPK pathways in *T. gondii*-induced VEGF production using various methods. PI3K/AKT pathway has been known for its role in maintaining various cell functions, including growth, migration, and survival (Aksamitiene et al., 2012). In the present study, *T. gondii* induced phosphorylation of AKT in ARPE-19 cells, and the treatment with PI3K inhibitors abolished VEGF expression. Our observations indicate that *T. gondii*-induced VEGF production in ARPE-19 cells is regulated by PI3K/AKT signaling pathway. Similar results have been reported in the studies that VEGF production is under regulation of PI3K/AKT as a result of protein kinase-R (PKR) down-regulaton or interferon-γ treatment in ARPE-19 cells (Liu et al., 2010; Zhu et al., 2016). MAPK signaling pathways are highly conserved and regulates diverse biological processes including proliferation, differentiation, survival, and stress responses (Aksamitiene et al., 2012). In this study, *T*. *gondii*-infected ARPE-19 cells had ERK1/2 and p38 MAPK activation, which is consistent with previous reports (Valere et al., 2003; Quan et al., 2015). And our study revealed that VEGF production was decreased dose-dependently by the treatment with PI3K, ERK1/2, and p38 MAPK inhibitors, but not with the JNK1/2 inhibitor. However, our results are different from a study in which hyperthermia was shown to induce temperature-dependent secretion of VEGF through p38, and to a lesser extent, JNK in the RPE cells (Faby et al., 2014). The presence of different pattern or signaling pathways for VEGF expression implies that the VEGF production regulation is a complex mechanism which involves multiple stimulants with various degree.

In addition, we attempted to identify the correlation between VEGF production and PI3K/MAPK signaling pathways in *T. gondii*-infected ARPE-19 cells. The experimental design includes adopting anti-VEGF agent or specific kinase inhibitors to inhibit the activities of VEGF or PI3K/MAPKs, and observation of the effect on each other's expression or phosphorylation, respectively. Anti-VEGF agents such as bevacizumab are highly effective for treating choroidal neovascularization (Ben Yahia et al., 2008). In this study, after the inhibition of VEGF activity by anti-VEGF agent or siRNA, the phosphorylation of ERK1/2 and AKT, but not p38 MAPK and JNK1/2, were significantly decreased in *T. gondii* infected ARPE-19 cells as compared to inhibitor untreated group. Interestingly, VEGF secretion was significantly reduced by pre-treatment with PI3K or ERK-1/2 inhibitors, but not with p38 MAPK or JNK1/2 inhibitors in *T. gondii*-infected group. These data suggest that there is a mutual interaction between the PI3K/AKT and ERK1/2 signaling pathways and the VEGF production in *T. gondii*-infected ARPE-19 cells. We also evaluated the relationship between VEGF and parasites. Inhibition of VEGF activity results in downregulation of intracellular proliferation of *T. gondii* tachyzoites in ARPE-19 cells, however, the invasion rate of *T. gondii* into ARPE-19 cells is not affected by VEGF. The role of host PI3K/AKT pathway in *T. gondii* proliferation but not in host infection or infiltration has been described in our previous work (Zhou et al., 2013). Our current results from *T. gondii* infection were also in line with those from Kruppel-like factor 4 (KLF4), which promotes angiogenesis by leading to VEGF expression, and subsequent enhancement of downstream pERK1/2 and pAKT signaling in human retinal microvascular endothelial cells (HRMECs) (Poon et al., 2015). Our findings with these supportive references might be helpful in understanding the pathophysiology of ocular toxoplasmosis and in finding a potential new therapeutic agent for treatment of ocular toxoplasmosis.

VEGF binds to its receptor kinases VEGF-R1, VEGF-R2 and VEGF-R3, and activates cellular signaling pathways (Claesson-Welsh, 2016). We evaluated the autocrine role and effect of VEGF signaling pathway in *T. gondii-*infected RPE on regulation of *T. gondii* induced PI3K/ERK1/2 pathway and parasite proliferation. Our results revealed that VEGF-R2 antagonist SU5416 inhibited VEGF production and tachyzoite proliferation dose-dependently, and reduced expressions of p-AKT and p-ERK1/2, even in the presence of *T. gondii*. These data imply that VEGF-R2 plays essential roles in both VEGF production and tachyzoite proliferation, and these process intermediated by the *T. gondii* induced regulation of AKT/ERK1/2 signaling pathways in RPE cells. Our findings are similar with the previous report describing that SU5416 inhibited VEGF and HIF-1α expression through the PI3K/AKT/p70S6K1 signaling pathway in ovarian cancer cells (Zhong et al., 2004). Collectively our observation revealed that in addition to the direct stimulation of host cell by parasite, secondary activation of host cell by autocrine factor, VEGF, might be indispensable for generating amplified and sustained signals to change and establish the *T. gondii*- favored host environment for its survival and proliferation. We also showed that PI3K and ERK1/2 pathways play essential role in the regulation of *T. gondii*-induced VEGF production and parasite proliferation in host cell.

**Figure 10:**
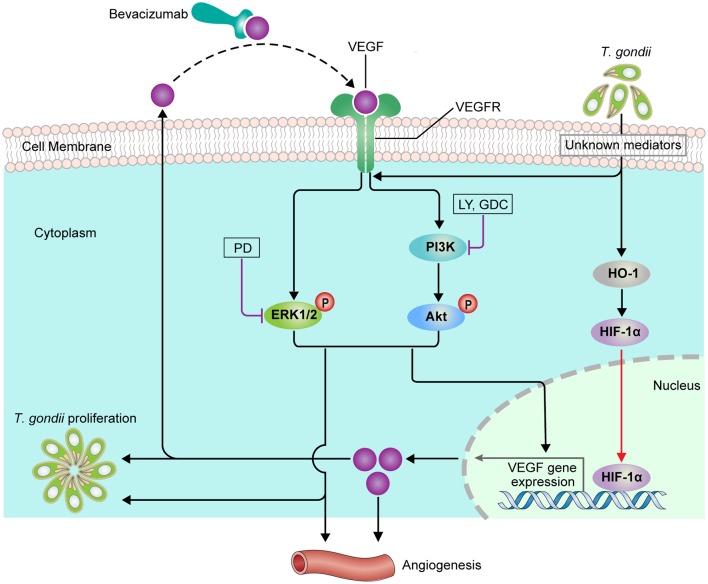
Scheme of functional relationship between *T. gondii*, VEGF and PI3K/MAPK signaling pathways in the parasite infected ARPE-19 cell.

We presume that at the early stage of infection, *T. gondii* stimulates pathogen sensors or receptors on host cell surface by directly binding or indirectly through its secretory molecules (e.g., ESAs) which will mediate the series activation of HO-1 and HIF-1α to initiate VEGF expression. Once this initial VEGF is secreted and activates host VEGF receptor in autocrine mode, the downstream signaling pathways (e.g., PI3K/MAPK pathways) will again induce the VEGF expression with more efficiency and with prolonged expression time (Figure 10). This series of event will form a positive feedback circuit to strengthen the whole signal to change host cell environment which will contribute in the *T. gondii* growth and replication and eventually in neovascularization in the *T. gondii-*infected tissue.

## Data Availability Statement

All datasets generated for this study are included in the article/Supplementary Material.

## Ethics Statement

The animal study was reviewed and approved by Animal experimental procedures were approved by the Institutional Animal Care and Use Committee (IACUC) at Chungnam National University (CNU-00706) and conformed to National Institutes of Health guidelines. The animals were fed standard rodent food and water ad libitum, and housed (maximum of 5 per cage) in sawdust-lined cages in an air-conditioned environment with 12-h light/dark cycles. Animal husbandry was provided by the staff of the IACUC under the guidance of supervisors who are certified Animal Technologists, and by the staff of the Animal Core Facility. Veterinary care was provided by IACUC faculty members and veterinary residents located on the Chungnam National University School of Medicine.

## Author Contributions

J-HQ, G-HC, and Y-HL conceived and designed the experiments. HI, J-HQ, Y-JJ, I-WC, FG, and J-QC performed the experiments. J-MY, J-HQ, and Y-HL analyzed the data. J-HQ and Y-HL wrote and critically revised the manuscript. All authors read and approved the final version of the manuscript.

## Conflict of Interest

The authors declare that the research was conducted in the absence of any commercial or financial relationships that could be construed as a potential conflict of interest.

## Supplementary Material

Supplementary Figure 1 Treatment with heat killed *T. gondii* has no effect on VEGF production in ARPE-19 cells. **(A)** ARPE-19 cells were treated with heat killed *T. gondii* at MOI of 1, 5 and 10 for 24 h and VEGF levels were evaluated using western blotting. **(B,C,D)** ARPE-19 cells were treated with heat killed *T. gondii* at various MOIs of 1 **(B)**, 5 **(C)** or 10 **(D)** for 0.5, 1, 18, and 24 h and the VEGF protein levels evaluated using western blotting.

Supplementary Figure 2 Formaldehyde fixed *T. gondii*-infection has no effect on VEGF production in ARPE-19 cells. **(A)** ARPE-19 cells were treated with formaldehyde fixed *T. gondii* at MOI of 1, 5, and 10 for 24 h and VEGF levels were evaluated using western blotting. **(B,C,D)** ARPE-19 cells were treated with formaldehyde fixed *T. gondii* at various MOIs of 1 **(B)**, 5 **(C)**, or 10 **(D)** for 0.5, 1, 18, and 24 h and the VEGF protein levels evaluated using western blotting.

Supplementary Figure 3 Viability of ARPE-19 cells after treatment with inhibitors of PI3K and MAPK signaling pathways. ARPE-19 cells were incubated with PI3K/AKT and MAPK inhibitors at the indicated doses for 2 h and their viabilities were assessed by MTT assay. Untreated cells served as control. LY294002, 1 and 10 μM; GDC-0941, 25 and 250 nM; PD098059, 3 and 30 μM; SB203580, 3 and 30 μM; SP600125, 3 and 30 μM. The bars represented the means and standard deviation of three independent experiments (*n*=3).

Supplementary Figure 4 Viability of ARPE-19 cells or *T. gondii* after treatment with anti-VEGF agent bevacizumab (BCM). ARPE-19 cells **(A)** or *T. gondii* tachyzoites **(B)** were incubated with BCM at the indicated doses for 24 or 48 h and their viabilities were assessed by MTT assay. Untreated cells served as control. The bars represented the means and standard deviation of three independent experiments (*n* = 3).
