# Supplementary material for: VEGF Production Is Regulated by the AKT/ERK1/2 Signaling Pathway and Controls the Proliferation of Toxoplasma gondii in ARPE-19 Cells
Source: Front Cell Infect Microbiol. 2020 Apr 28;10:184. doi: 10.3389/fcimb.2020.00184 (PMC7216739; doi:10.3389/fcimb.2020.00184)
Supplement: Supplementary Figure 1 — Treatment with heat killed T. gondii has no effect on VEGF production in ARPE-19 cells. (A) ARPE-19 cells were treated with heat killed T. gondii at MOI of 1, 5 and 10 for 24 h and VEGF levels were evaluated using western blotting. (B,C,D) ARPE-19 cells were treated with heat killed T. gondii at various MOIs of 1 (B), 5 (C) or 10 (D) for 0.5, 1, 18, and 24 h and the VEGF protein levels evaluated using western blotting. [file Presentation_1.PPTX]

## Slide 1
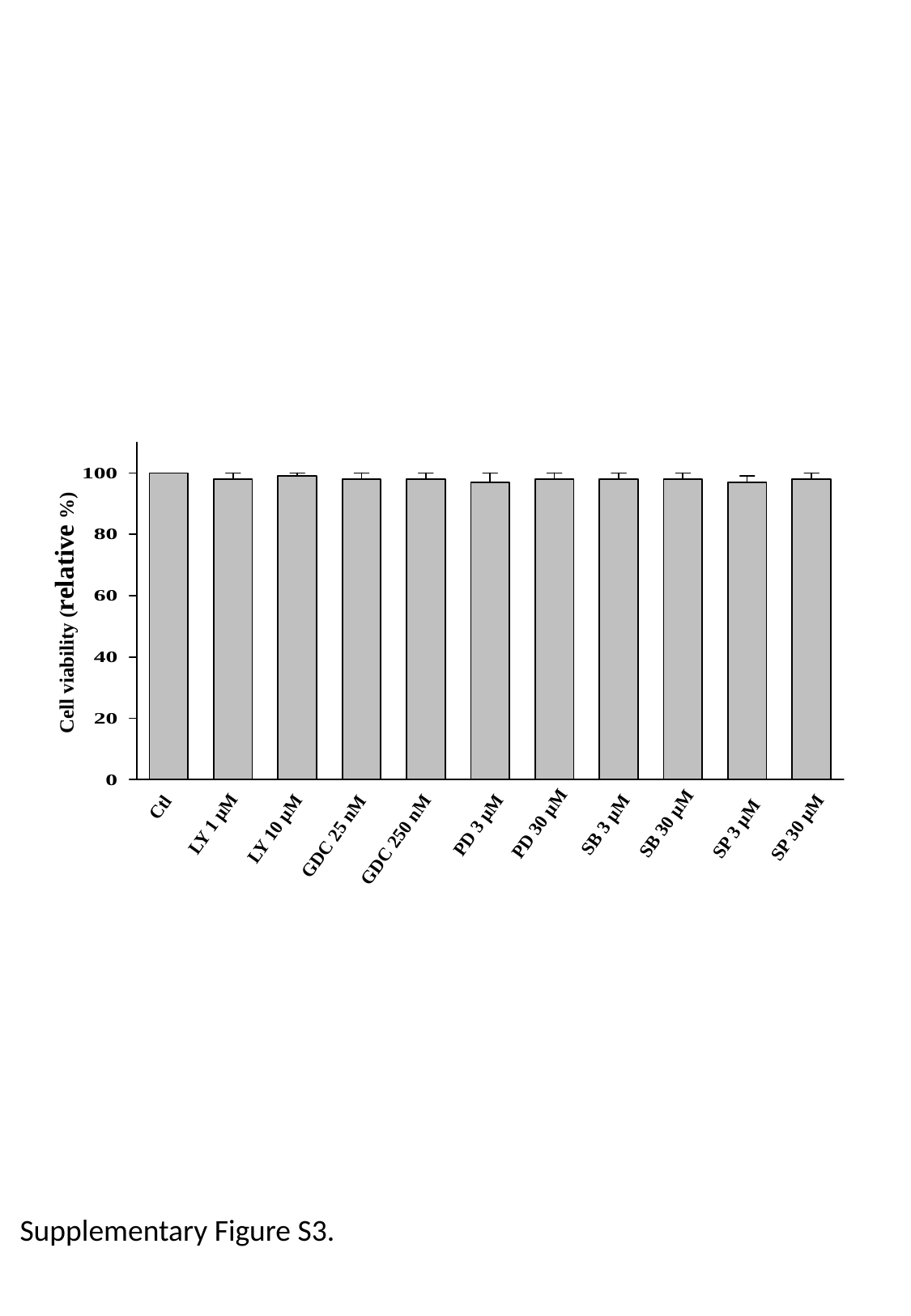

Cell viability (relative %)
Ctl
SB 30 µM
PD 30 µM
LY 1 µM
SB 3 µM
PD 3 µM
SP 30 µM
SP 3 µM
LY 10 µM
GDC 25 nM
GDC 250 nM
Supplementary Figure S3.
