# Supplementary material for: VEGF Production Is Regulated by the AKT/ERK1/2 Signaling Pathway and Controls the Proliferation of Toxoplasma gondii in ARPE-19 Cells
Source: Front Cell Infect Microbiol. 2020 Apr 28;10:184. doi: 10.3389/fcimb.2020.00184 (PMC7216739; doi:10.3389/fcimb.2020.00184)
Supplement: Supplementary Figure 2 — Formaldehyde fixed T. gondii-infection has no effect on VEGF production in ARPE-19 cells. (A) ARPE-19 cells were treated with formaldehyde fixed T. gondii at MOI of 1, 5, and 10 for 24 h and VEGF levels were evaluated using western blotting. (B,C,D) ARPE-19 cells were treated with formaldehyde fixed T. gondii at various MOIs of 1 (B), 5 (C), or 10 (D) for 0.5, 1, 18, and 24 h and the VEGF protein levels evaluated using western blotting. [file Presentation_2.PPTX]

## Slide 1
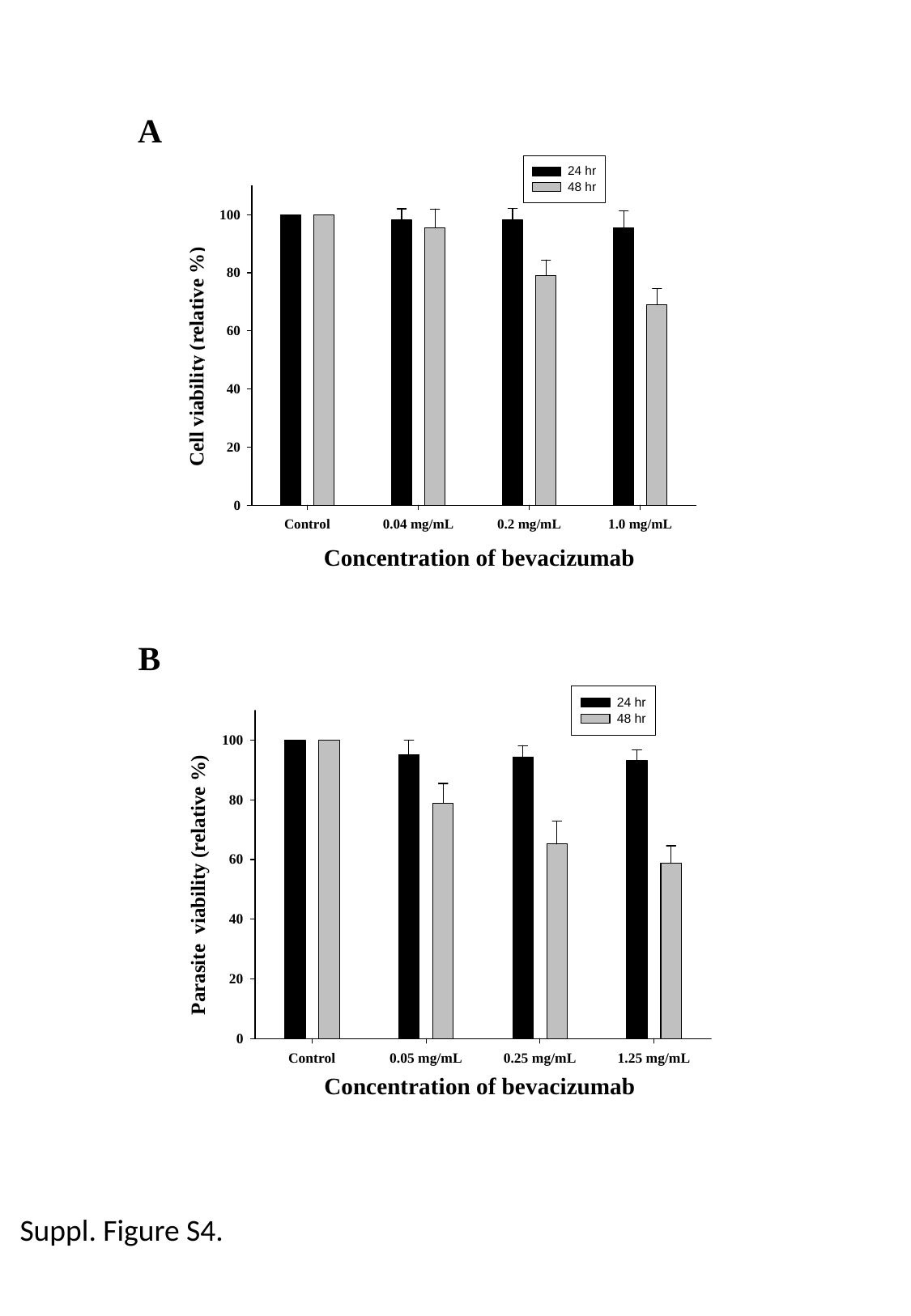

A
Cell viability (relative %)
Concentration of bevacizumab
B
Parasite viability (relative %)
Concentration of bevacizumab
Suppl. Figure S4.
