# Supplementary figures and images for: VEGF Production Is Regulated by the AKT/ERK1/2 Signaling Pathway and Controls the Proliferation of Toxoplasma gondii in ARPE-19 Cells
Source: Front Cell Infect Microbiol. 2020 Apr 28;10:184. doi: 10.3389/fcimb.2020.00184 (PMC7216739; doi:10.3389/fcimb.2020.00184)

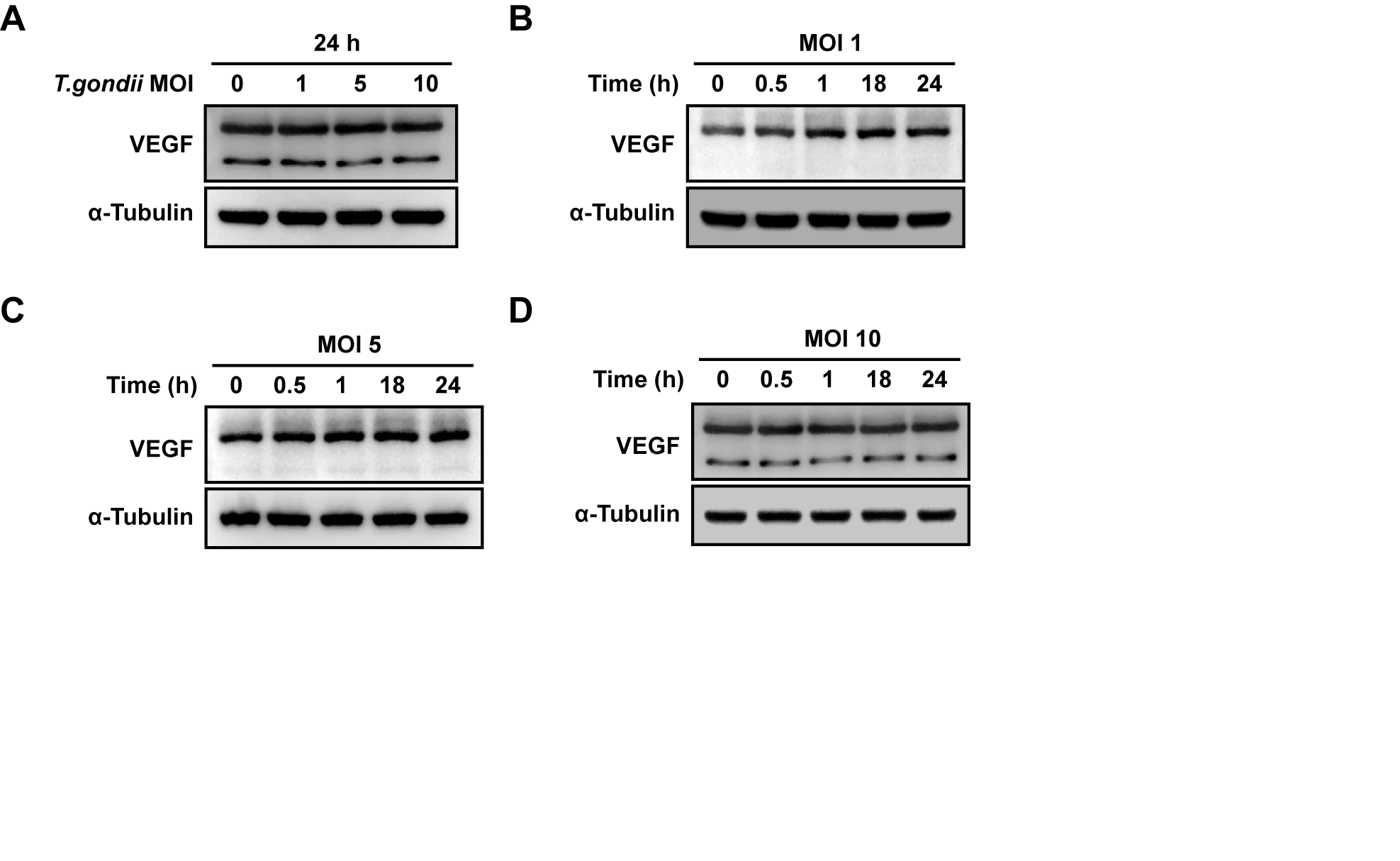

Supplement: Supplementary Figure 3 — Viability of ARPE-19 cells after treatment with inhibitors of PI3K and MAPK signaling pathways. ARPE-19 cells were incubated with PI3K/AKT and MAPK inhibitors at the indicated doses for 2 h and their viabilities were assessed by MTT assay. Untreated cells served as control. LY294002, 1 and 10 μM; GDC-0941, 25 and 250 nM; PD098059, 3 and 30 μM; SB203580, 3 and 30 μM; SP600125, 3 and 30 μM. The bars represented the means and standard deviation of three independent experiments (n=3). [file Image_1.TIF]

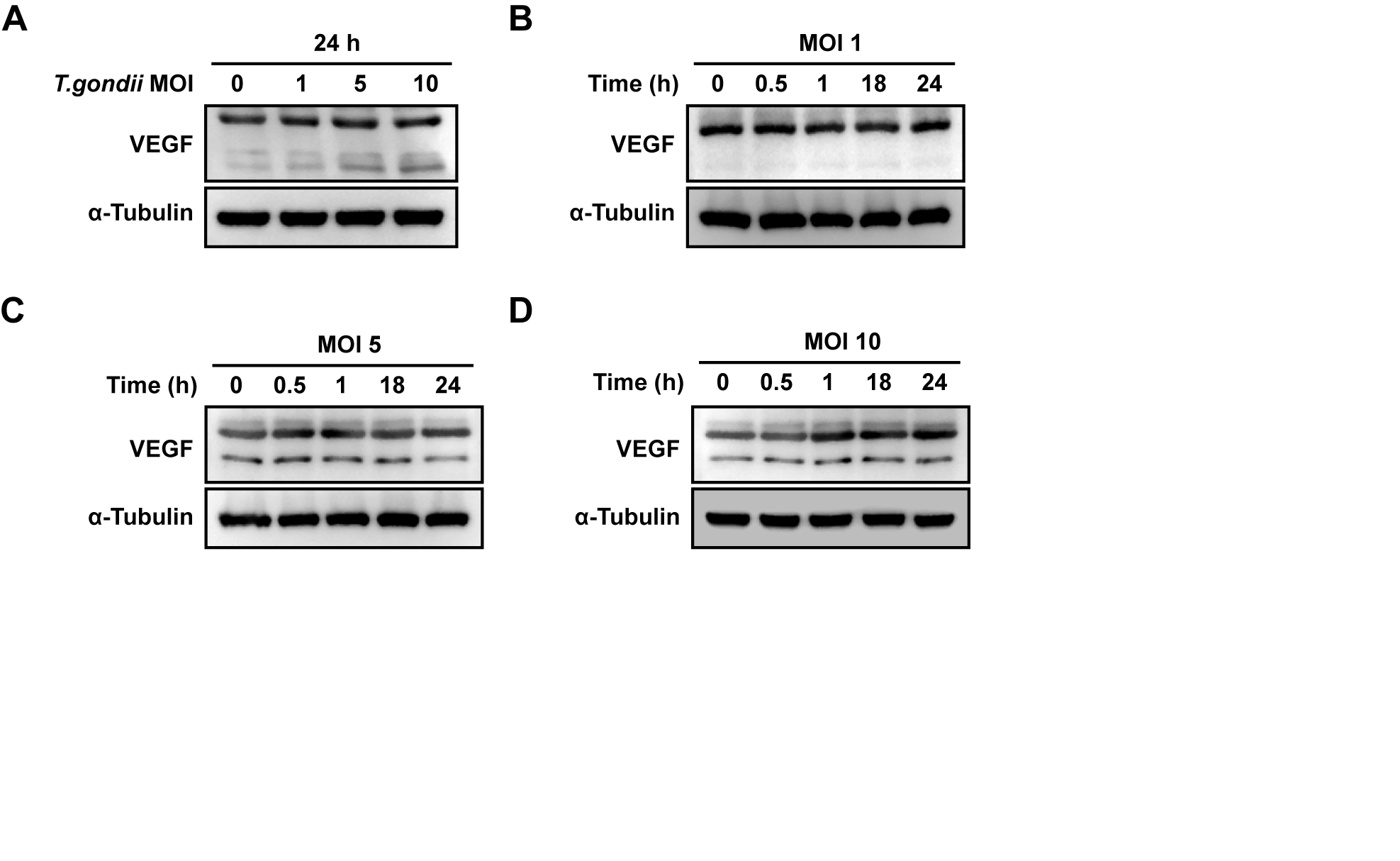

Supplement: Supplementary Figure 4 — Viability of ARPE-19 cells or T. gondii after treatment with anti-VEGF agent bevacizumab (BCM). ARPE-19 cells (A) or T. gondii tachyzoites (B) were incubated with BCM at the indicated doses for 24 or 48 h and their viabilities were assessed by MTT assay. Untreated cells served as control. The bars represented the means and standard deviation of three independent experiments (n = 3). [file Image_2.TIF]
